# Tumor volumes as a predictor of response to the anti-EGFR antibody drug conjugate depatuxizumab mafadotin

**DOI:** 10.1093/noajnl/vdab102

**Published:** 2021-08-03

**Authors:** Hui K Gan, Sagun Parakh, Andrew B Lassman, Aidan Seow, Eddie Lau, Sze Ting Lee, Malaka Ameratunga, Yuliya Perchyonok, Diana Cao, Ingrid J G Burvenich, Graeme J O’Keefe, Angela Rigopoulos, Erica Gomez, David Maag, Andrew M Scott

**Affiliations:** 1 Tumour Targeting Program, Olivia Newton-John Cancer Research Institute, Austin Health, Heidelberg, Melbourne, Australia; 2 La Trobe University School of Cancer Medicine, Heidelberg, Melbourne, Australia; 3 Department of Medical Oncology, Austin Health, Heidelberg, Melbourne, Australia; 4 Department of Medicine, University of Melbourne, Parkville, Australia; 5 Department of Medical Oncology, Monash Health, Clayton, Melbourne, Australia; 6 Division of Neuro-Oncology, Department of Neurology, Columbia University Vagelos College of Physicians and Surgeons, Herbert Irving Comprehensive Cancer Center, NewYork-Presbyterian Hospital, New York, NY, USA; 7 Department of Molecular Imaging and Therapy, Austin Health, Heidelberg, Melbourne, Australia; 8 Department of Radiology, Austin Health, Heidelberg, Melbourne, Australia; 9 Research and Development Department, AbbVie Inc., North Chicago, Illinois, USA

**Keywords:** depatuxizumab mafadotin, GBM, tumor volume

## Abstract

**Background:**

The adverse impact of increasing brain tumor size on the efficacy of antibody-drug conjugates (ADCs) was investigated preclinically then validated with clinical data.

**Methods—Preclinical study:**

The impact of tumor size on ADC tumor delivery and treatment response was evaluated in an *EGFR*-amplified patient-derived glioblastoma (GBM) model following treatment with Depatuxizumab mafadotin (Depatux-M). Biodistribution and imaging studies correlated drug distribution with starting treatment volume and anti-tumor activity.

**Methods—Clinical study:**

M12-356 was a Phase I study of Depatux-M in patients with GBM. Blinded volumetric analysis of baseline tumor volumes of M12-356 patients was undertaken by two reviewers and results correlated with response and survival.

**Results:**

Preclinically, imaging and biodistribution studies showed specific and significantly higher tumor uptake of zirconium-89 labeled Depatux-M (^89^Zr-Depatux-M) in mice with smaller tumor volume (~98 mm^3^) versus those with larger volumes (~365 mm^3^); concordantly, mice with tumor volumes ≤100 mm^3^ at treatment commencement had significantly better growth inhibition by Depatux-M (93% vs 27%, *P* < .001) and significantly longer overall survival (*P* < .0001) compared to tumors ≥400 mm^3^. Clinically, patients with tumor volumes <25 cm^3^ had significantly higher response rates (17% vs. 0%, *P* = .009) and longer overall survival (0.5 vs 0.89 years, *P* = .001) than tumors above 25 cm^3^.

**Conclusion:**

Both preclinical and clinical data showed intra-tumoral concentration and efficacy of Depatux-m inversely correlated with tumor size. This finding merit further investigation with pretreatment tumor volume as a predictor for response to ADCs, in both gliomas and other solid tumors.

**Key Points**

- Tumor volumes directly correlated to ADC tumour uptake and efficacy in preclinical models and in humans with brain tumors.
- Future trials of ADCs may consider restriction of eligibility and/or stratification by tumor volume in the study design.

**Importance of the Study**

Little significant improvement in survival of GBM patients has been made in more than a decade. Many recent trials have investigated antibody and antibody-drug conjugates (ADCs) with disappointing results, often despite success in other tumor types. We show that tumor size directly impacts the deliverable drug concentration of ADCs in brain tumors, and tumor size has a significant impact on therapeutic response. As tumor size can be modulated through surgical or drug approaches, investigating this phenomenon further has implications for the development and use of all large therapeutic molecules in brain tumor patients. In addition, these findings extend beyond GBM as they may be applicable to ADCs and in other tumor types.

There is increasing interest in the use of antibodies and antibody-based constructs in glioblastoma (GBM) trials.^1^ As antibodies are large macromolecules, with a molecular weight of approximately 150 kDaltons,^2^ barriers exist to penetration of brain tumors, even where there are breaches of the blood-brain barrier as seen in enhancing tumors of high-grade gliomas.^3^ Concomitantly, other factors such as drug polarity and the presence of complex transport mechanisms may further impede drug penetration. Increased interstitial pressure observed in large tumors is likely one barrier. As GBMs increase in size, larger interstitial pressures develop due to high tumor cell density, increased vascular permeability, and impaired lymphatics.^4^ This results in heterogeneous concentrations of drugs in different regions of the tumor.^5^ Furthermore, other factors in larger tumors which adversely affect drug uptake including dysfunctional vascular networks and increasing areas of hypoperfusion.^6^

We investigated the impact of tumor volume on treatment outcomes using the antibody-drug conjugate (ADC), Depatuxizumab mafodotin (Depatux-M previously ABT-414, AbbVie), both preclinically and clinically. Depatux-M comprises a tumor-specific epidermal growth factor receptor (EGFR) targeting antibody (Depatux, formerly mAb806) linked to the cytotoxin monomethyl auristatin F (MMAF). Importantly, the unconjugated antibody ABT-806 showed no conventional toxicities associated with inhibitors of EGFR signaling, like rash and diarrhea, and imaging of biodistribution of ^111^In-ABT-806 showed no normal tissue uptake, highlighting the tumor-specific characteristics of the targeting antibody.^7–9^ Preclinical data showed in vivo activity of Depatux-M in tumor models with overexpression of wild-type EGFR, *EGFR* amplification, or EGFRvIII mutation.^10^ In the three-arm phase I study (M12-356 study, NCT01800695), Depatux-M given concurrently with radiation therapy and temozolomide in patients with newly diagnosed GBM had an acceptable toxicity profile.^11^ In the randomized phase II study, INTELLANCE 2/EORTC 1410,^12^ patients with first recurrence GBM were randomised to Depatux-M with or without temozolomide, or lomustine only, or temozolomide only depending on the time of relapse. In this study, the combination of Depatux-M with temozolomide had a 1-year OS rate of 40% versus 28% with lomustine or temozolomide only (HR 0.68, *P* = .024). No OS difference was observed between Depatux-M monotherapy and temozolomide or lomustine (median OS 7.9 months).

To investigate the impact of tumor size on Depatux-M therapeutic activity, we undertook biodistribution studies using zirconium-89 labeled Depatux-M (^89^Zr-Depatux-M) to quantitate drug concentration in large and small volume GBMs. We then correlated tumor size with growth inhibition in vivo following Depatux-M treatment. To validate our preclinical findings, we undertook a volumetric analysis of baseline tumor volumes in M12-356 patients and correlated results with patient response as the reduction in drug uptake in larger tumors could be reasonable expected to impact tumor response to Depatux-M. This differential response rate should also be reflected in patient survival, so we undertook to examine the relationship between tumor size and survival.

## Methods

### Preclinical Study

**Figure 1.**
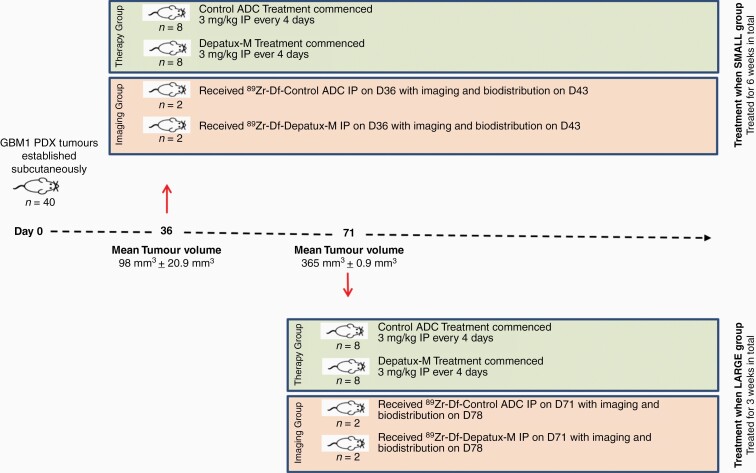
Study schema of in vivo bioimaging and therapy study.

GBM1 tumors were established subcutaneously in NSG mice (Figure 1). Mice were divided into two groups of 20 each with either small (98 mm^3^ ± 20.9 mm^3^) or large (365 mm^3^ ± 0.9 mm^3^) tumors. In each group, 8 mice received treatment with either Depatux-M or an isotype IgG control ADC, and 2 mice were imaged with either ^89^Zr-Depatux-M or ^89^Zr-ADC-control (see Figure 1 and Supplementary Material). Mice were injected with ^89^Zr-Df-Depatux-M or the ^89^Zr-Df-control ADC respectively on the same day as mice in the therapy group commenced treatment with Depatux-M (Figure 1). Mice from the small size group received between 39–56.2 µg, 41–62.4 µCi ^89^Zr-Df-control ADC (*n* = 2), or ^89^Zr-Df- Depatux-M (*n* = 2) in 100 µl, via tail vein injection. Mice from the large size group received between 38.7–55.3 µg, 30.6–53.2 µCi ^89^Zr-Df-ADC-control (*n* = 2) or ^89^Zr-Df-Depatux-M (*n = 2*) via tail vein injection. ^89^Zr uptake in normal tissues and tumors was then assessed using PET imaging and MRI performed on a NanoScan PET/MRI (Mediso, Hungary) at the ACRF Centre for Translational Cancer Therapeutics and Imaging (Melbourne, Australia). For biodistribution, all mice were humanely sacrificed by isoflurane over-inhalation after the 168-hour imaging time point and tissues collected for assessment. All animal studies were approved by the Austin Health Animal Ethics Committee and were conducted in compliance with the Australian Code (8th Edition 2013) for the care and use of animals for scientific purposes.

### Clinical Study

To confirm our preclinical data, three reviewers (EL and AS as primary with adjudication as needed by AMS) undertook an independent volumetric analysis of baseline tumor volumes in brain MRI scans of patients treated with Depatux-M on the M12-356 study (Supplementary Figure 2). Manual segmentation of the MRI scans was performed using MIM Maestro^TM^ (MIM Software Inc, Cleveland, OH) under the direction of experienced neuro-oncological radiologists (EL, YP) using 5mm slice thickness of post-gadolinium T1 weighted images (T1wGd) and FLAIR sequences. Precontrast T1 sequences reviewed but not segmented. All nonartefactual FLAIR abnormalities, including suspected edema, were segmented on the FLAIR sequence, with only enhancing disease segmented on T1wGd. The surgical cavity, cysts, and necrosis were not included as per RANO criteria.^13^ In cases with multiple disease foci, all regions of interest were segmented. For each case, volumetric data on T1wGd and FLAIR images were documented. Responses were assessed per the RANO criteria in the original M12-356 study, which in brief, incorporate at least 50% reduction in cross-sectional area of contrast enhancement as part of definition of response and at least 25% increase for progressive disease. All clinical data were obtained as part of a clinical trial (NCT01800695) approved by a Human Ethics Committee.

### Statistical Analysis

Data from in vivo experiments were analyzed using one-way ANOVA with posthoc Bonferroni correction for TGImax and the Mantel–Cox log-rank test for survival. Analyses were performed using Prism® Version 8.0 (GraphPad, CA). All *P*-values are two-sided and values ≤.05 were considered significant.

Response rates were compared using chi-square and Fisher’s exact tests. Inter-rater correlation and agreement between measurements were assessed using Intraclass correlation coefficient (ICC).^14^ ICC estimates were calculated using SPSS version 25 (SPSS Inc. Chicago, IL), two-way mixed-effects, single rater, consistency model. Overall survival was measured from the date of registration to the date of death from any cause. Kaplan-Meier estimates of OS from commencement of therapy were calculated separately for each group and compared using a log-rank test, where *P*-values ≤ .05 were considered statistically significant.

## Results

### Therapy Study with ^89^Zr-labeled Depatux-M

Half the mice commenced treatment on day 36 when the average tumor volume was <100 mm^3^ (98 ± 20.9 mm^3^) (Treatment at Small Size group). Depatux-M caused substantial and significant tumour growth inhibition (TGI) compared to control ADC (36.72 ± 6.14 mm^3^ vs. 511.02 ± 71.6 mm^3^; *P* < .001) with a TGImax of 93%. This result was consistent with multiple previous experiments using these drugs in this model, and other GBM models.^10^ The remaining mice were treated on day 71 when their tumor size was 365 mm^3^ (Treatment at Large Size group). In this group, Depatux-M also caused significant growth inhibition compared to control ADC but of a lesser magnitude than in smaller tumors (434.95 ± 62.44 mm^3^ vs. 625.21 ± 67.28 mm^3^; *P* < .01); TGImax was only 27%, which was significantly less than 93% observed in the Treatment at Small Size group (*P* < .001).

### Bioimaging and Biodistribution Studies with ^89^Zr-labeled Depatux-M

**Figure 2.**
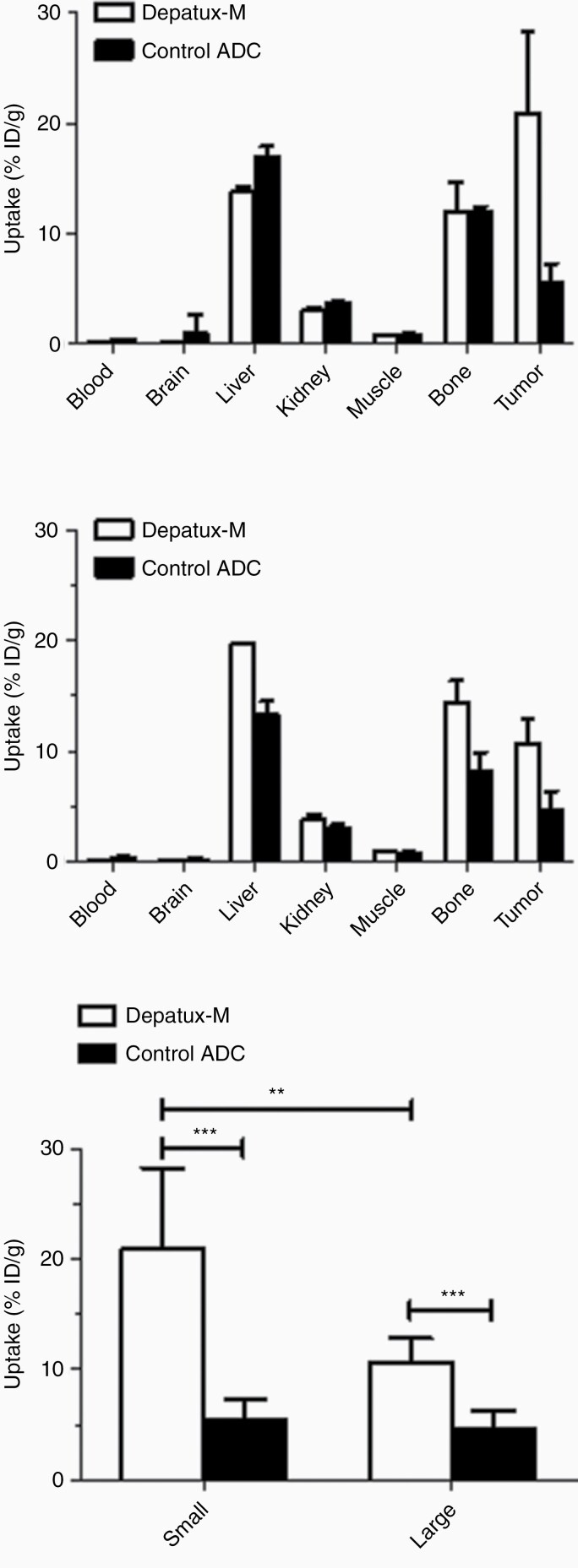
Biodistribution of ^89^Zr-Df- Depatux-M compared to ^89^Zr-Df-control ADC in vivo. Biodistribution of ^89^Zr-Df-Depatux-M in NSG mice bearing GBM patient derived xenografts on day 7 postinjection (bars; mean ± SD; *n* = 8); (A) Small tumor group (*n* = 8) and (B) Large tumour group (*n* = 6). (C) Tumor uptake of ^89^Zr-Df-Depatux-M on day 7 postinjection (bars; mean ± SD) in the small and large tumor groups versus control. **, *P* < .01; ***, *P* < .001.

To investigate why the Large Size group showed a smaller TGI than the Small Size group, a combined imaging and biodistribution study was performed to investigate drug uptake at the smaller and larger tumor sizes. Mice were imaged on day 0, day 3, and day 7 postinjection of ^89^Zr-Depatux-M or ^89^Zr-Control ADC. Radioconjugates were successfully produced with the end of synthesis radiochemical purity > 98%, and high immunoreactivity for antigen positive U87MG.de2-7 cells with ^89^Zr-Df-Depatux-M (92.27 ± 6.50%). After the final imaging time point on Day 7, all mice were sacrificed and tumor uptake of ^89^Zr-Depatux-M and ^89^Zr-Control ADC were measured. Biodistribution data on Day 7 shows that ^89^Zr-Control ADC uptake in both the Small Size and Large Size group were low and not significantly different (5.49% ± 1.77 ID/g versus 4.69 ± 1.58 %ID/g respectively, *P* = .5054, Figure 2A and 2C). By contrast, tumor uptake of ^89^Zr-Depatux-M was significantly higher in the Smaller versus the Larger size group (20.93 ± 7.11 ID/g versus 10.67 ± 2.34 %ID/g respectively, *P* = .0047). In all groups, uptake in liver and bone was consistent with previous literature of ^89^Zr-Df metabolism in mice.^15^

**Figure 3.**
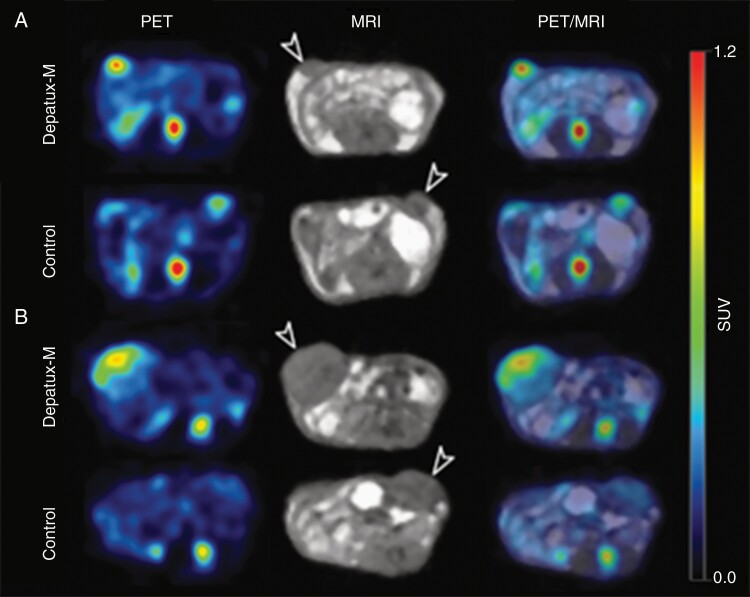
PET/MR imaging with 89Zr-Df-ABT-414-ADC and 89Zr-Df-isotope control in (A) small versus (B) large tumours on D3 postinjection. White arrows indicate location of the tumours. Maximal uptake in the small tumors (A, PET image) is higher than that in the large tumors (B, PET image) confirmed on quantitative analysis.

Whole-body PET/MR images confirmed the biodistribution results, showing a higher uptake of ^89^Zr-Depatux-M in the tumors of the Smaller versus Larger size group (Figure 3A and 3B respectively). Similarly, to the biodistribution data, the corresponding bioimaging study whole-body PET/MR images showed higher uptake of ^89^Zr-Depatux-M in Smaller versus Larger size group (13.22 ± 5.27%ID/g versus 6.54 ± 1.11 %ID/g). By contrast, ^89^Zr-Control ADC uptake in both the Small Size and Large Size group were low and not significantly different (5.61% ± 0.83 %ID/g versus 4.71 ± 1.58 %ID/g respectively).

### Clinical Study

***Patient characteristics***

The M12-356 study (NCT01800695) was an open-label, phase I, 3-arm dose-escalation and expansion study: Arm A evaluated Depatux-M concurrently with radiation and temozolomide (TMZ) in newly diagnosed GBM, Arm B evaluated Depatux-M with TMZ in newly diagnosed GBM as adjuvant therapy as well as in recurrent GBM and Arm C evaluated Depatux-M as monotherapy in recurrent GBM (Supplementary Figure 2).^16–18^

We obtained baseline MRI scans for all M12-356 patients and undertook a volumetric analysis of tumor volumes at study entry. For Arm A patients, these were the post-operative and preradiotherapy MRI scans. A total of 202 patients were included in the current analysis. Baseline demographic data are detailed in Table 1. The median age was 57 years, majority were male (59%) and majority (87%) had a Karnofsky performance scale ≥ 80. Tumors were *EGFR* amplified in 71% of patients and *EGFRvIII* mutant in 52%. Only 13% of those tested were *MGMT* promoter methylated.

**Table 1.** Baseline Demographics

Demographics Characteristics	*N* (%)
All patients	202 (100)
Median age—years (range)	(20–80)
Sex	
Male	124 (59)
Female	78 (37)
Karnofsky Performance	
70	22 (11)
80	60 (29)
90	79 (38)
100	41 (20)
EGFR amplification status	
Amplified	148 (71)
Not amplified	45 (21)
Unknown	9 (4)
*EGFRvIII* mutation status	
Positive	110 (52)
Negative	82 (39)
Unknown	10 (5)
MGMT methylation status	
Methylated	28 (13)
Unmethylated	59 (28)
Unknown	115 (55)
Treatment arm A—Adjuvant	
(Depatux-M in combination with radiation and temozolomide)	45 (22)
Arm A: Dose escalation	24 (11)
Arm A: Dose expansion	21 (10)
Treatment arm B—Recurrent	
(Depatux-M in combination with temozolomide)	68 (34)
Arm B: Dose escalation	15 (7)
Arm B: Dose expansion	53 (25)
Treatment arm B—Adjuvant	14 (7)
Arm B: Adjuvant, Dose escalation	14 (7)
Treatment arm C—Recurrent	
(Depatux-M monotherapy)	75(36)
Arm C: Monotherapy	75 (36)

EGFR, Epidermal growth factor receptor; MGMT, O6-methylguanine-DNA methyltransferase.

***Efficacy***We showed that there was excellent inter-rater correlation for volumetric analysis using T1wGd and FLAIR images (kappa = 0.89, *P* < .0001 and kappa = 0.97, *P* < .0001 respectively). Importantly, we found that patients with *EGFR* amplified recurrent GBMs (*n* = 110) treated with Depatux-M, either alone (Arm C) or in combination with TMZ (Arm B), had significantly more responders in patients with tumor volumes < 25 cm^3^ compared to those ≥25 cm^3^ at study entry (response rate of 17% vs 0%, *P* = .009 two-sided). In fact, no patient with a ≥25 cm^3^ had a RANO response. When analyzing by arm, responses were also more frequent in smaller than larger tumors in Arm B (50 patients who received Depatux-M with temozolomide, response rate 24% vs 0%, *P* = .089 two-sided) and Arm C (60 patients who received Depatux-M alone, response rate 10% vs 0%, *P* = .287) but did not reach statistical significance in this underpowered posthoc exploratory subset analysis. Of note, most patients in this study had recently progressed (within 6 months) on TMZ and the likelihood of response to TMZ alone in this patient population would be low.^17^ Patients in Arm A were not considered evaluable for response due to recent completion of radiotherapy.

**Figure 4.**
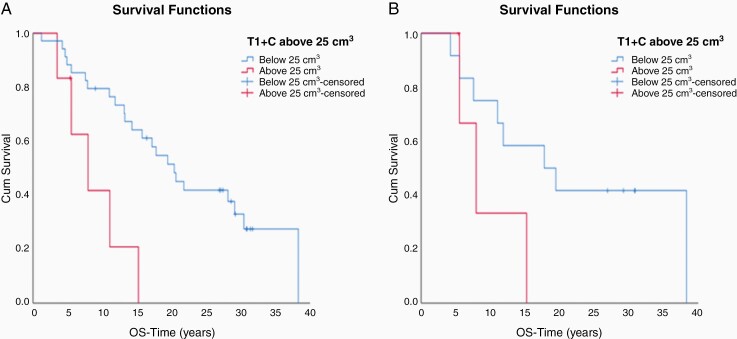
(A) Patients with newly diagnosed GBM on Arms A and B with tumor volumes below 25 cm^3^ had a significantly longer median OS than those above 25 cm^3^ (2.0 vs 0.8 years; *P* = .006 respectively). (B) Patients on Arms A and B with EGFR amplified tumor volumes below 25 cm^3^ had improved survival than those above 25 cm^3^ (1.8 vs 0.8 years respectively, *P* = .28)

**Figure 5.**
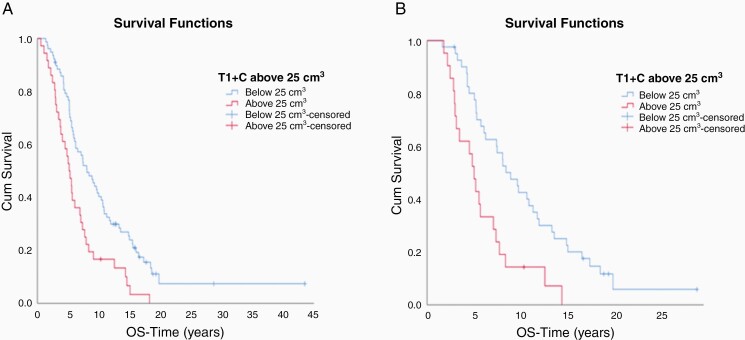
(A) Patients with rGBM on Arms B and C with tumor volumes below 25 cm^3^ had a significantly longer median OS than those above 25 cm^3^ (0.81 vs 0.52 years respectively; *P* = .001). (B) Patients with rGBM on Arm C with tumor volumes below 25 cm^3^ had significantly improved survival than those above 25 cm^3^ (0.89 vs 0.50 years respectively, *P* = .001)

We also examined the impact of tumor volume on survival. Patients with newly diagnosed GBM (*n* = 41) enrolled on arms A and B whose tumor volumes were below 25 cm^3^ (post-operatively but before chemoradiotherapy), had a significantly longer median OS than those above 25 cm^3^ (2.0 vs 0.8 years; *P* = .006) respectively) (Figure 4A). In this cohort, analysis in the subset of patients with EGFR *amplified* tumors (*n* = 16) trended toward improved survival for patients with tumors volumes <25 cm^3^ than > 25 cm^3^ (1.8 vs 0.8 years, *P* = .28) (Figure 4B). Patients in Arms B and C with *EGFR* amplified recurrent GBMs (*n* = 110) with tumors < 25 cm^3^ had a longer median survival than patients with tumors ≥25 cm^3^ (0.81 vs 0.52 years, *P* = .001) (Figure 5A). This association of tumor volume and survival was also seen in patients on Arm C (*n* = 60), with tumor volumes < 25 cm^3^ significantly associated with longer OS (0.89 vs 0.50 years, *P* = .001) (Figure 5B).

## Discussion

To our knowledge, this is the first study to quantify the impact of tumor volume on the uptake, retention, and therapeutic efficacy of ADCs in brain tumors. Data to date would suggest that large tumors in other sites are associated with increased interstitial pressure, abnormal vasculature and lymphatics, increased rates of necrosis, and increase heterogeneity.^4–6,19^ For example interstitial pressures in center of large tumors may be four-fold higher than for small tumors.^19^ Our results strongly support our hypothesis that larger tumor size, with associated adverse features expected to reduce drug penetrance and uptake, resulted in significant reductions in the uptake of Depatux-M in preclinical models, with corresponding reduction in tumor growth inhibition. Clearly, this raises the possibility of the potential confounding influence of tumor volume on assessment of efficacy of Depatux-M. However, these findings have implications for the optimal integration of all ADCs or other large molecules in the management of GBM, strongly supporting strategies that would reduce tumor size and/or interstitial pressure to increase their efficacy. Furthermore, tumor size is rarely controlled for in many GBM trials and may contribute to underestimation of drug efficacy.

Several strategies to mitigate the impact of larger tumors are potentially available. Bevacizumab results in vascular normalization, reduces vascular permeability, and has been shown to impact intra-tumoral drug distribution and potentially efficacy of ADCs when used in combination.^20^ For example, platinum-resistant epithelial ovarian cancer xenograft models treated with the ADC Mirvetuximab soravtansine (IMGN853), a FRα-binding antibody linked to a tubulin-disrupting maytansinoid (DM4), in combination with bevacizumab resulted in significant tumor regression that was superior to either bevacizumab or the ADC alone in ovarian cancer.^21^ It was postulated the presence of bevacizumab results in better tumor penetration and exposure to the ADC, resulting in more effective eradication of tumor cells. These findings support investigating anti-angiogenic and ADC combinatorial approaches to further enhance the therapeutic benefit of these agents. The ongoing phase Ib FORWARD II trial (NCT02606305) is evaluating the Mirvetuximab soravtansine in combination with bevacizumab in pts with platinum-resistant ovarian cancer.^22^ Another approach would be simple debulking of the tumor prior to ADC treatment. Surgery at recurrence has shown to be associated with a survival advantage and has not shown to significantly affect the quality of life.^23–26^ With evolving surgical techniques and better patient selection, more patients are then able to receive systemic therapy postoperatively.^25^ A number of other experimental pharmacological and physical strategies have been investigated to reduce tumoral interstitial pressure including imatinib, paclitaxel, dexamethasone, angiotensin II, TGFβ inhibitors as well as hyperthermia, radiotherapy, photodynamic therapy, and focused ultrasound therapy.^27,28^

Clearly, these findings require further validation, ideally in orthotopic models and including other drug treatment, followed by a prospective clinical trial if appropriate. We are currently undertaking additional work to investigate how tumor volume relates to traditional prognostic and predictive biomarkers in this nonrandomized phase 1 study. We are also currently seeking to confirm our findings with data from Depatux-M in the INTELLANCE 1 and 2 studies of Depatux-M in newly diagnosed and recurrent GBM, respectively, where the randomized designs would allow us to differentiate between the predictive impact of tumor volume on survival based on differential tumor efficacy compared to the well-known prognostic effects of tumor volume.^29–32^ These analyses will provide us with definitive data as to whether tumor volume impacts drug delivery and patient outcomes. If our initial findings are confirmed, it will require a significant change in how future research and clinical trials are designed.

## Conclusion

Increased tumor volumes result in significant reduction in ADC penetration in GBM preclinically and response in patients. The impact of this as a modifiable factor, within the broader prognostic impact of increased tumor volume, warrants further investigation with prospective and/or randomized trials.

## Supplementary Material

vdab102_suppl_Supplementary_Figure_S1

vdab102_suppl_Supplementary_Figure_S2

vdab102_suppl_Supplementary_Materials

## Notes

**Conflict of interest statement**. H.G. has had consulting roles and research funding with AbbVie; speakers bureau and travel support from Ignyta, honoraria from Merck, BMS and Eisai. D.M., H.-J.L., E.G. are full-time employees of AbbVie and may own stock. A.M.S. has consulting roles and research funding from EMD Serono, Abbvie, Astra Zeneca, Telix, Medimmune; he is consultant to Life Science Pharmaceuticals; he has patents on mAb806. A.B.L. (last 2 years) had honoraria and associated travel support from Novocure, Karyopharm, Sapience, Abbott, QED, Society for Neuro-Oncology, Italian Foundation For Cancer Research, Forma, Bayer, Orbus, Bioclinica as an expert blinded independent reviewer of clinical and imaging data for a BMS-sponsored trial, NW Bio, ASCO, AbbVie, Physicians’ Education Resource/Chemotherapy Foundation Symposium, Agios.

## Notes

**Authorship Statement**. Experiments and analysis were performed by E.L., A.S., S.P., A.R., I.B., G.O., and D.C., A.B.L., A.M.S., and H.K.G. supervised the project; S.P. and H.K.G. wrote the paper and all other authors critically reviewed it; all authors read and approved the final manuscript.

## References

[1] Kaplon H, Reichert JM. Antibodies to watch in 2019. Mabs. 2019;11(2):219–238. 30516432 10.1080/19420862.2018.1556465 PMC6380461

[2] Weiner LM, Surana R, Wang S. Monoclonal antibodies: versatile platforms for cancer immunotherapy. Nat Rev Immunol. 2010;10(5):317–327. 20414205 10.1038/nri2744 PMC3508064

[3] Sarkaria JN, Hu LS, Parney IF, et al. Is the blood–brain barrier really disrupted in all glioblastomas? A critical assessment of existing clinical data. Neuro Oncol. 2017;20(2):184–191. 10.1093/neuonc/nox175 PMC5777482 29016900

[4] Leu AJ, Berk DA, Lymboussaki A, Alitalo K, Jain RK. Absence of functional lymphatics within a murine sarcoma: a molecular and functional evaluation. Cancer Res. 2000;60(16):4324–4327. 10969769

[5] Munson JM, Shieh AC. Interstitial fluid flow in cancer: implications for disease progression and treatment. Cancer Manag Res. 2014;6:317–328. 25170280 10.2147/CMAR.S65444 PMC4144982

[6] Harder BG, Blomquist MR, Wang J, et al. Developments in blood-brain barrier penetrance and drug repurposing for improved treatment of glioblastoma. Front Oncol. 2018;8:462. 30406029 10.3389/fonc.2018.00462 PMC6206841

[7] Cleary JM, Yee LK-C, Azad N, et al. Abstract 2506: a phase 1 study of ABT-806, a humanized recombinant anti-EGFR monoclonal antibody, in patients with advanced solid tumors. Cancer Res. 2012;72(8 Supplement):2506-2506.

[8] Luwor RB, Johns TG, Murone C, et al. Monoclonal antibody 806 inhibits the growth of tumor xenografts expressing either the de2-7 or amplified epidermal growth factor receptor (EGFR) but not wild-type EGFR. Cancer Res. 2001;61(14):5355–5361. 11454674

[9] Gan HK, Burgess AW, Clayton AH, Scott AM. Targeting of a conformationally exposed, tumor-specific epitope of EGFR as a strategy for cancer therapy. Cancer Res. 2012;72(12):2924–2930. 22659454 10.1158/0008-5472.CAN-11-3898

[10] Phillips AC, Boghaert ER, Vaidya KS, et al. ABT-414, an antibody drug conjugate targeting a tumor-selective EGFR epitope. Mol Cancer Ther. 2016;15:661–669. 26846818 10.1158/1535-7163.MCT-15-0901

[11] Reardon DA, Lassman AB, van den Bent M, et al. Efficacy and safety results of ABT-414 in combination with radiation and temozolomide in newly diagnosed glioblastoma. Neuro Oncol. 2016;19:965–975. 10.1093/neuonc/now257 PMC5570193 28039367

[12] van den Bent M, Eoli M, Sepulveda JM, et al. INTELLANCE 2/EORTC 1410 randomized phase II study of Depatux-M alone and with temozolomide vs temozolomide or lomustine in recurrent EGFRamplified glioblastoma. Neuro Oncol. 2019;22(5):684–693. 10.1093/neuonc/noz222 PMC7229258 31747009

[13] Wen PY, Macdonald DR, Reardon DA, et al. Updated response assessment criteria for high-grade gliomas: response assessment in neuro-oncology working group. J Clin Oncol. 2010;28(11):1963–1972. 20231676 10.1200/JCO.2009.26.3541

[14] Koo TK, Li MY. A guideline of selecting and reporting intraclass correlation coefficients for reliability research. J Chiropr Med. 2016;15(2):155–163. 27330520 10.1016/j.jcm.2016.02.012 PMC4913118

[15] Sharma SK, Chow A, Monette S, et al. Fc-Mediated anomalous biodistribution of therapeutic antibodies in immunodeficient mouse models. Cancer Res. 2018;78(7):1820–1832. 29363548 10.1158/0008-5472.CAN-17-1958 PMC5882577

[16] Gan HK, Reardon DA, Lassman AB, et al. Safety, pharmacokinetics, and antitumor response of depatuxizumab mafodotin as monotherapy or in combination with temozolomide in patients with glioblastoma. Neuro Oncol. 2017;20(6):838–847. 10.1093/neuonc/nox202 PMC5961429 29077941

[17] Lassman AB, van den Bent MJ, Gan HK, et al. Safety and efficacy of depatuxizumab mafodotin+ temozolomide in patients with EGFR-amplified, recurrent glioblastoma: results from an international phase I multicenter trial. Neuro Oncol. 2018;21(1):106–114. 10.1093/neuonc/noy091 PMC6303422 29982805

[18] Lassman AB, Bent MJVD, Gan HK, et al. Efficacy analysis of ABT-414 with or without temozolomide (TMZ) in patients (pts) with EGFR-amplified, recurrent glioblastoma (rGBM) from a multicenter, international phase I clinical trial. J Clin Oncol. 2017;35(15_suppl):2003-2003.

[19] Jain RK. Physiological barriers to delivery of monoclonal antibodies and other macromolecules in tumors. Cancer Res. 1990;50(3 Suppl):814s–819s. 2404582

[20] Goel S, Duda DG, Xu L, et al. Normalization of the vasculature for treatment of cancer and other diseases. Physiol Rev. 2011;91(3):1071–1121. 21742796 10.1152/physrev.00038.2010 PMC3258432

[21] Ponte JF, Ab O, Lanieri L, et al. Mirvetuximab Soravtansine (IMGN853), a folate receptor alpha-targeting antibody-drug conjugate, potentiates the activity of standard of care therapeutics in ovarian cancer models. Neoplasia. 2016;18(12):775–784. 27889646 10.1016/j.neo.2016.11.002 PMC5126132

[22] O’Malley DM, Martin LP, Gilbert L, et al. Mirvetuximab soravtansine, a folate receptor alpha (FRα)-targeting antibody-drug conjugate (ADC), in combination with bevacizumab in patients (pts) with platinum-resistant ovarian cancer: Maturing safety and activity profile from the FORWARD II phase 1b study. J Clin Oncol. 2018;36(15_suppl):5549-5549.

[23] McGirt MJ, Chaichana KL, Gathinji M, et al. Independent association of extent of resection with survival in patients with malignant brain astrocytoma. J Neurosurg. 2009;110(1):156–162. 18847342 10.3171/2008.4.17536

[24] Yong RL, Wu T, Mihatov N, et al. Residual tumor volume and patient survival following reoperation for recurrent glioblastoma. J Neurosurg. 2014;121(4):802–809. 25061868 10.3171/2014.6.JNS132038

[25] Parakh S, Thursfield V, Cher L, et al. Recurrent glioblastoma: Current patterns of care in an Australian population. J Clin Neurosci. 2016;24:78–82. 26549675 10.1016/j.jocn.2015.08.025

[26] Wann A, Tully PA, Barnes EH, et al. Outcomes after second surgery for recurrent glioblastoma: a retrospective case-control study. J Neurooncol. 2018;137(2):409–415. 29294233 10.1007/s11060-017-2731-2

[27] Heldin CH, Rubin K, Pietras K, Ostman A. High interstitial fluid pressure - an obstacle in cancer therapy. Nat Rev Cancer. 2004;4(10):806–813. 15510161 10.1038/nrc1456

[28] Ariffin AB, Forde PF, Jahangeer S, Soden DM, Hinchion J. Releasing pressure in tumors: what do we know so far and where do we go from here? A review. Cancer Res. 2014;74(10):2655–2662. 24778418 10.1158/0008-5472.CAN-13-3696

[29] Iliadis G, Kotoula V, Chatzisotiriou A, et al. Volumetric and MGMT parameters in glioblastoma patients: survival analysis. BMC Cancer. 2012;12:3. 22214427 10.1186/1471-2407-12-3 PMC3264493

[30] Bette S, Barz M, Wiestler B, et al. Prognostic value of tumor volume in glioblastoma patients: size also matters for patients with incomplete resection. Ann Surg Oncol. 2018;25(2):558–564. 29159745 10.1245/s10434-017-6253-0

[31] Gorlia T, Stupp R, Brandes AA, et al. New prognostic factors and calculators for outcome prediction in patients with recurrent glioblastoma: a pooled analysis of EORTC Brain Tumour Group phase I and II clinical trials. Eur J Cancer. 2012;48(8):1176–1184. 22464345 10.1016/j.ejca.2012.02.004

[32] Grant R, Walker M, Hadley D, Barton T, Osborn C. Imaging response to chemotherapy with RMP-7 and carboplatin in malignant glioma: size matters but speed does not. J Neurooncol. 2002;57(3):241–245. 12125987 10.1023/a:1015768225145

