# Supplementary figures and images for: Tumor volumes as a predictor of response to the anti-EGFR antibody drug conjugate depatuxizumab mafadotin
Source: Neurooncol Adv. 2021 Aug 3;3(1):vdab102. doi: 10.1093/noajnl/vdab102 (PMC8446913; doi:10.1093/noajnl/vdab102)

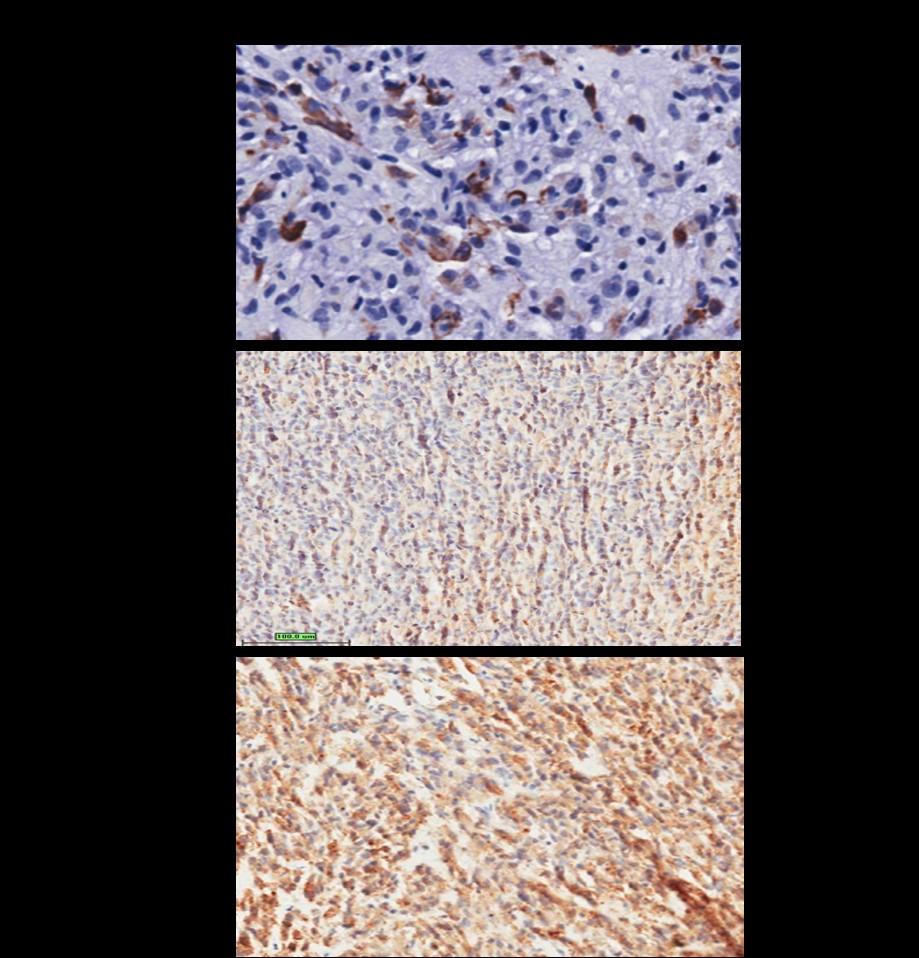

Supplement: vdab102_suppl_Supplementary_Figure_S1 [file vdab102_suppl_supplementary_figure_s1.jpeg]

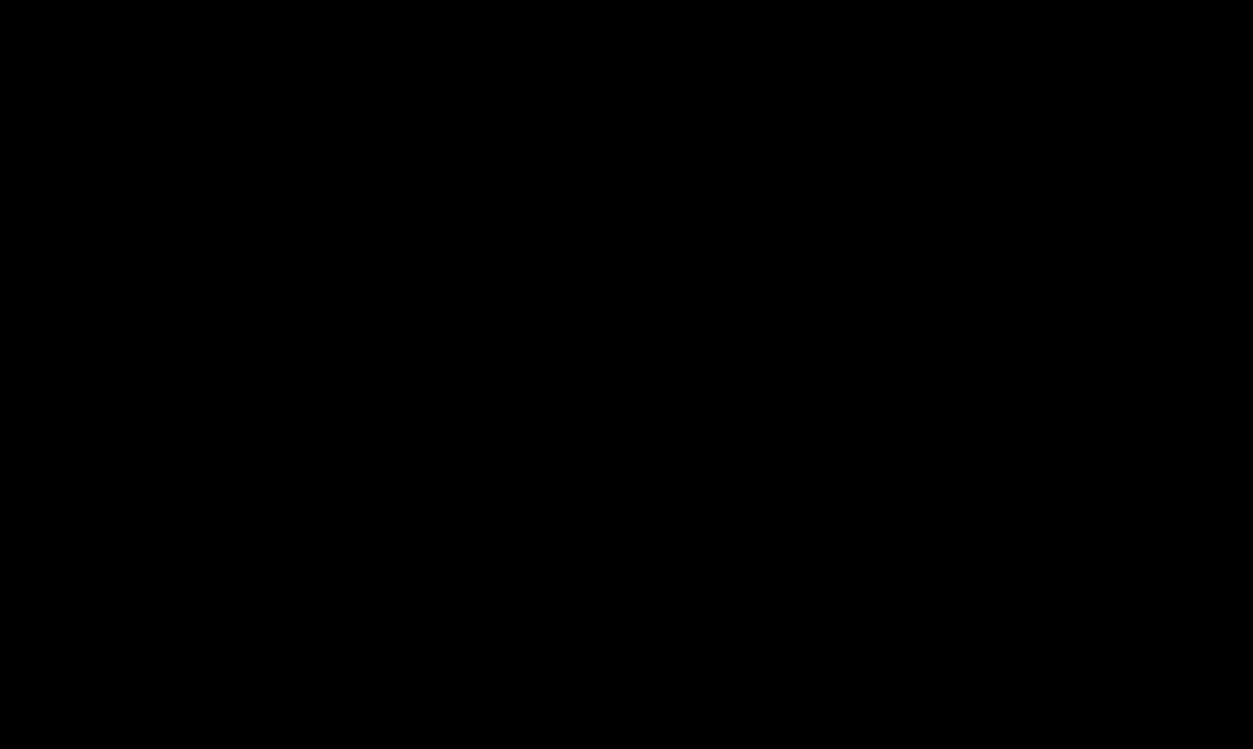

Supplement: vdab102_suppl_Supplementary_Figure_S2 [file vdab102_suppl_supplementary_figure_s2.jpeg]
