# Supplementary material for: Tumor volumes as a predictor of response to the anti-EGFR antibody drug conjugate depatuxizumab mafadotin
Source: Neurooncol Adv. 2021 Aug 3;3(1):vdab102. doi: 10.1093/noajnl/vdab102 (PMC8446913; doi:10.1093/noajnl/vdab102)
Supplement: vdab102_suppl_Supplementary_Materials [file vdab102_suppl_supplementary_materials.docx]

**Supplementary Figure I:** GBM1 is a patient derived xenograft (PDX) cell line established using tumor samples from a male patient and shown to be GFAP positive, EGFR amplified and EGFRvIII positive cell line

**Supplementary Figure II:** M12-356 study schema

**Supplementary Data**

The Mediso PET-MR has a specification spatial resolution of 0.9 mm, however due to the average positron kinetic energy for ^89^Zr (402.7 keV)^1^ compared to ^18^F (252 keV),^1^ a Partial-Volume-Effect (PVE) characterization measurement was performed. For ^89^Zr, this results in a positron range of 1.27 mm compared to 0.66 mm for ^18^F.^1^ A series of measurements using a solution of ^89^Zr with a concentration of 1 kBq / µL were undertaken utilizing volumes ranging from 5 µL to 1000 µL.

The resultant Recovery-Coefficient (RC) curve was parameterized to allow the RC value to be determined for a given lesion volume. Lesion volumes ranged from 20 µL to 1000 µL with corresponding RC’s of 28% to 100%.


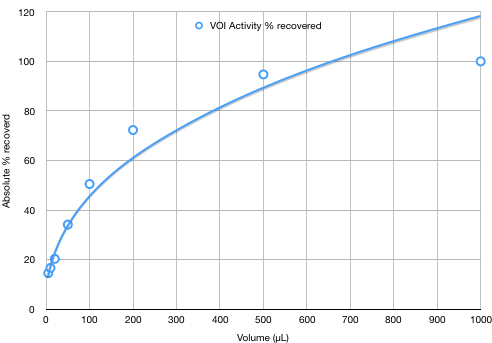


Figure I. Measured Recovered Activity

Based on the ^89^Zr range determined by Jødal,^1^ the resultant Mediso PET/MR system resolution for ^89^Zr is calculated to be 1.5 mm. In order to account for the loss of localization of uptake due to the positron range, a deconvolution algorithm was applied to the data. The Richardson-Lucy ^2,3^ algorithm was applied and the optimal parameters were found to be a 3d-gaussian with 2 mm FWHM with 5 iterations. An example deconvolution processing is given in Figure 2.


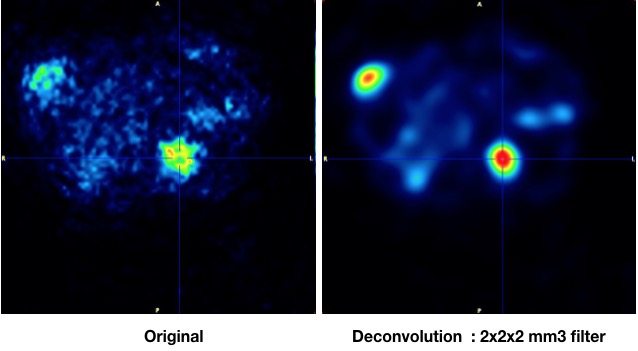


Figure II. Transaxial mouse section: Original Mediso reconstruction and Richardson-Lucy Deconvolved image

GBM1 is a patient derived xenograft (PDX) cell line established from an M12-356 patient at clinical relapse and confirmed to express glial fibrillary acidic protein (GFAP) and harbor *EGFR* amplification and *EGFRvIII* mutation, consistent with the primary patient tumor phenotype (see Supplementary Figure 1).

Treatment with Departux-M in preclinical models utilised a previously established optimal dose of 3 mg/kg intraperitoneally (IP) every 4 days.^4^ Tumor volume (V) was measured two to three times weekly. Measurements of the length (L) and width (W) of the tumor were obtained via callipers and the volume was calculated according to the equation: V = L × W^2^/2. Mice were euthanized when the average tumor volume was >1000 mm^3^ or if mice displayed prolonged symptoms of stress. Tumor growth inhibition (TGImax) was calculate to determine the difference between the mean tumor volume of the treated group and the control group.

For the imaging studies, radiolabeling of Depatux-M and control ADC with zirconium-89 (^89^Zr, Austin Health Cyclotron, Heidelberg, VIC, Australia) was performed via the bifunctional metal ion chelator *p*-isothiocyanatobenzyldesferrioxamine (Df; Macrocyclics Inc Dallas, TX, USA), according to methods previously described.^5^ Radioconjugates were synthesized on the day of administration. Radiochemical purity was assessed by instant thin-layer chromatography (iTLC) and immunoreactivity for antigen positive cells assessed as previously described.^6^

During imaging with ^89^Zr-Df-Depatux-M or the ^89^Zr-Df-control ADC, mice were anesthetised with inhalational isoflurane then transferred to an anaesthesia / imaging closed chamber for whole body MRI (T1-weighted) followed by a 30-minute PET static scan. Scans were done at 2, 72 and 168 hours after injection. All PET raw data were decay, dead time, random and attenuation corrected and reconstructed into volumetric images with a transaxial matrix size 255 x 255 using the built–in quasi–Monte Carlo stimulation algorithm combined with stochastic iteration and filtered sampling. The voxel dimension in the reconstructed images was 0.4 x 0.4 x 0.4mm. The image-based biodistribution analysis consisted of defining volumes of interest (VOI) from the acquired MRI images. Tumour volumes of interest (mL) were determined for each time point based on mark-up of tumours in cross-sectional MRI images. These VOIs were applied to the co-registered PET image data from which the lesion volume and the tracer uptake radioactivity were determined. Whole body volumes were determined from MR images on day 0 for all mice and whole body average activities (kBq/mL) determined from co-registered PET images. Counts of the tumours were converted to percentage injected dose/g (%ID/g) using the whole body activity values obtained on day 0. The whole body values obtained on day 0 were decay corrected for day 3 and day 7 images. For simplicity, 1 mL of tumour volume was considered equal to 1 g of tumour. The measured lesion uptake activity was corrected for the effects of partial volume effect (PVE)

For the biodistribution study, blood was collected by cardiac puncture, and tumour and tissues were collected immediately and counted for radioactivity together with injected dose standards in a dual-channel γ-scintillation counter (Wizard; PerkinElmer). Triplicate standards prepared from the injected material were counted at each time point with tissue and tumor samples, enabling calculations to be corrected for physical decay of the isotope. The tissue distribution data were calculated as the mean ± SD percentage injected dose per gram tissue (%ID/g) for the radiolabelled construct per time point.

References

**1.** Jødal L, Le Loirec C, Champion C. Positron range in PET imaging: an alternative approach for assessing and correcting the blurring. *Phys Med Biol.* 2012; 57(12):3931-3943.

**2.** Richardson WH. Bayesian-based iterative method of image restoration. *JoSA.* 1972; 62(1):55-59.

**3.** Holmes TJ, Liu YH. Richardson-Lucy/maximum likelihood image restoration algorithm for fluorescence microscopy: further testing. *Appl Opt.* 1989; 28(22):4930-4938.

**4.** Reilly EB, Phillips AC, Boghaert ER, et al. ABT-414, an antibody drug conjugate targeting a tumor-selective EGFR epitope. *Mol Cancer Ther.* 2016; 15:661-669.

**5.** Burvenich IJG, Parakh S, Lee FT, et al. Molecular imaging of T cell co-regulator factor B7-H3 with (89)Zr-DS-5573a. *Theranostics.* 2018; 8(15):4199-4209.

**6.** Panousis C, Rayzman VM, Johns TG, et al. Engineering and characterisation of chimeric monoclonal antibody 806 (ch806) for targeted immunotherapy of tumours expressing de2-7 EGFR or amplified EGFR. *Br J Cancer.* 2005; 92(6):1069-1077.
